# Supplementary material for: Study by Optical Spectroscopy of Bismuth Emission in a Nanosecond-Pulsed Discharge Created in Liquid Nitrogen
Source: Molecules. 2021 Dec 6;26(23):7403. doi: 10.3390/molecules26237403 (PMC8658815; doi:10.3390/molecules26237403)
Supplement: Supplementary file 1 [file molecules-26-07403-s001.zip › molecules-1464348-supplementary file corrected.pdf]

SUPPLEMENTARY FILE

Study by Optical Spectroscopy of Bismuth Emission in a Nanosecond-Pulsed Discharge Created in Liquid Nitrogen

Anna Nominé[1], Cédric Noel[1], Thomas Gries[1], Alexandre Nominé[1,2], Valentin Milichko[2] and Thierry Belmonte[1]

[1] Université de Lorraine, CNRS, IJL, F-54000 Nancy, France

[2] ITMO University, St. Petersburg 197101, Russia

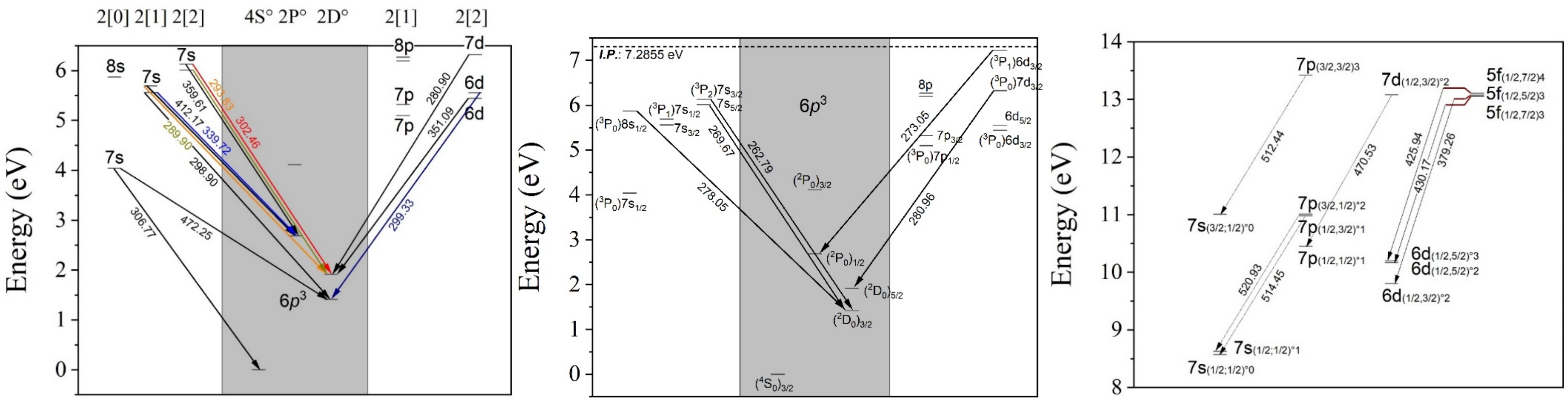

Figure S1: Bi I and Bi II Grotrian diagrams and observed optical transitions.

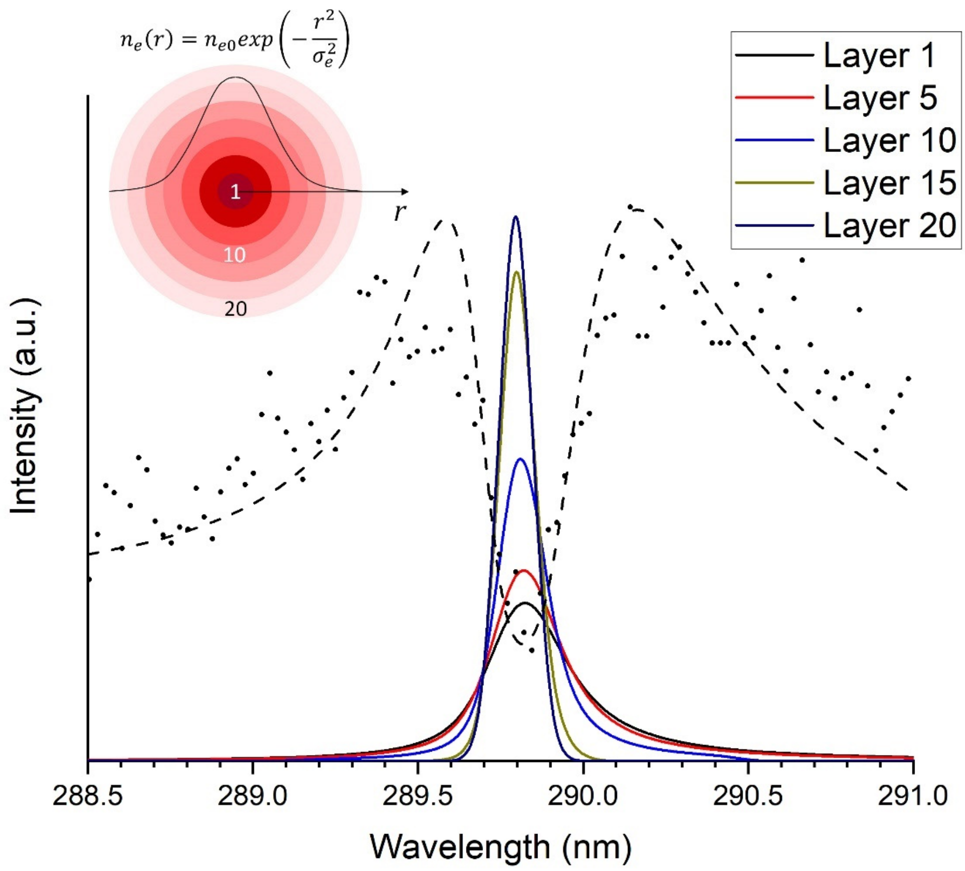

Figure S2: Emission intensities of the transition at 289.80 nm in 5 chosen layers of the optical model leading to the profile given by the dashed line. Dots are experimental data. Photons at central wavelength are trapped by atoms in the lower level and take longer to escape the medium, leading to a hole in the distribution. We do verify that emission lines become narrower and symmetrical as the electron density decreases from layer 1 to layer 20.

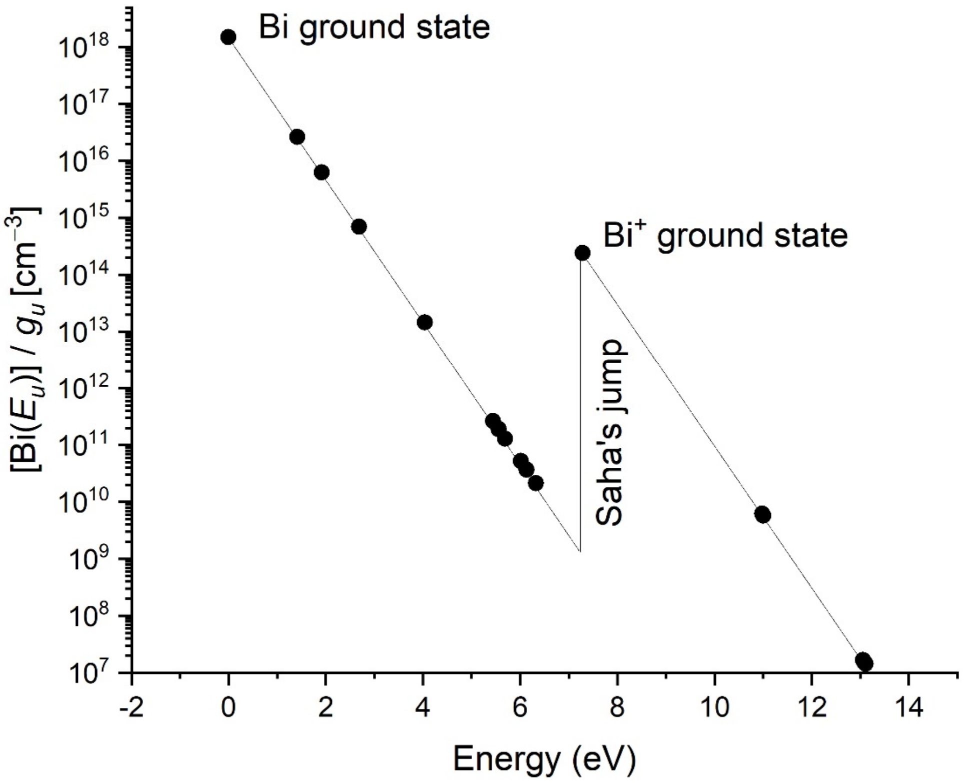

Figure S3: Boltzmann plot showing the distribution in energy of the Bi I and Bi II states identified in this work.

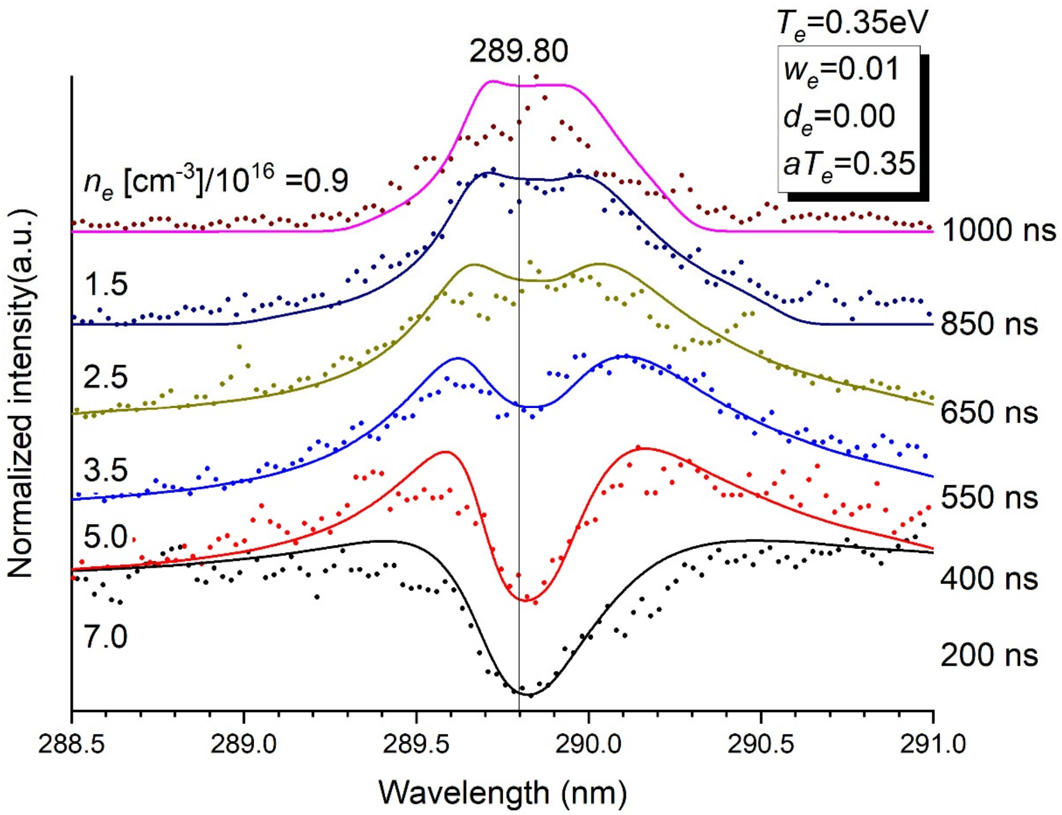

Figure S4a: Normalized intensity of the emission of the transition at 289.80 nm as a function of time. Stark parameters are given the inset. The electron density n_e in [cm^{-3}]/10^{16} is determined for each time. T_e = 0.35 eV. P = 5 bars.

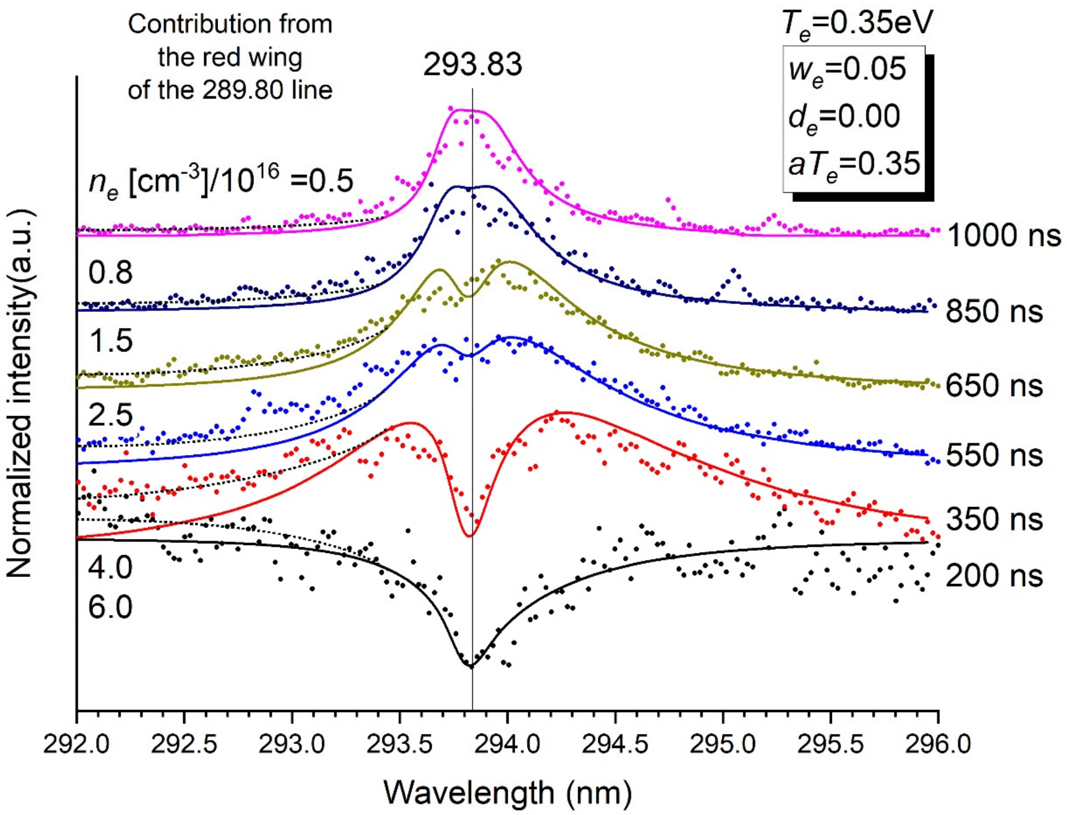

Figure S4b: Normalized intensity of the emission of the transition at 293.83 nm as a function of time. Stark parameters are given the inset. The blue part of the line is perturbed by the red wing of the 289.80 transition, which must be included for a correct description of that part of the spectrum. The electron density n_e in [cm^{-3}]/10^{16} is determined for each time. T_e = 0.35 eV. P = 5 bars.

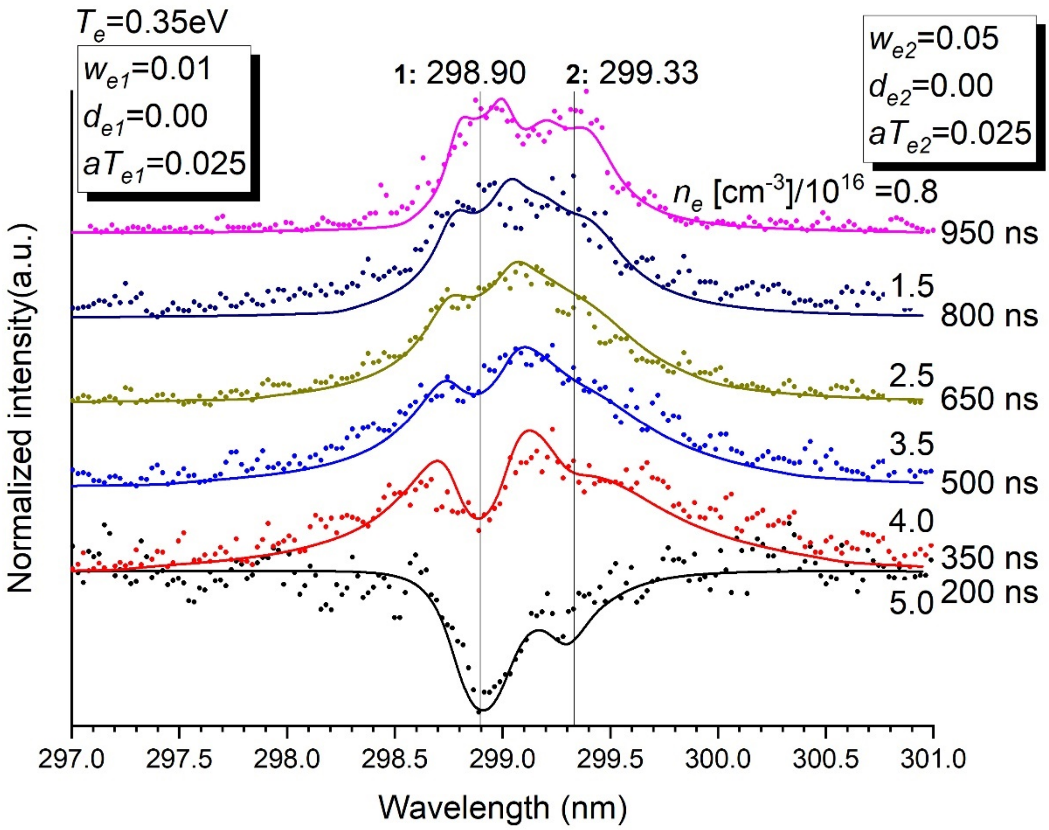

Figure S4c: Normalized intensity of the emission of the transitions at 298.90 nm and 299.33 nm as a function of time. Stark parameters for each transition are given the insets. The electron density n_e in [cm^{-3}]/10^{16} is determined for each time. T_e = 0.35 eV. P = 5 bars.

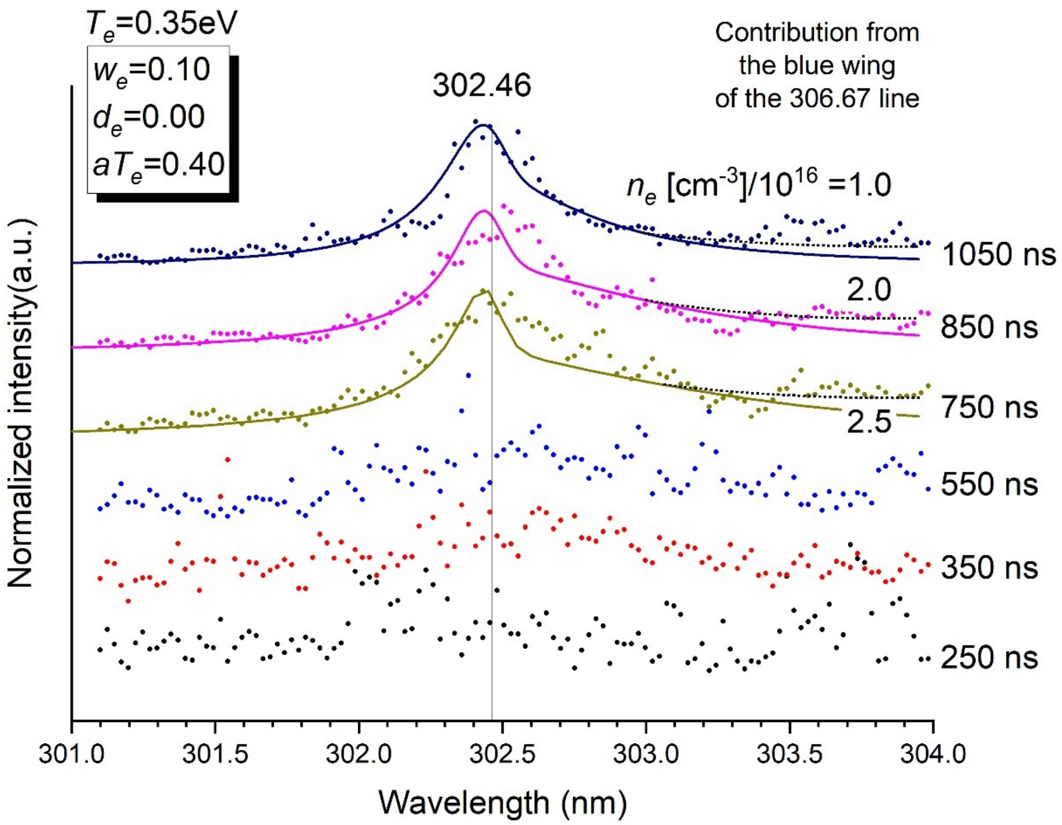

Figure S4d: Normalized intensity of the emission of the transition at 302.46 nm as a function of time. Stark parameters are given the inset. The red part of the line is perturbed by the blue wing of the 306.67 transition, which must be included for a correct description of that part of the spectrum. Data at 250, 350 and 550 ns are too noisy to be exploited. The electron density n_e in [cm^{-3}]/10^{16} is determined for each time. T_e = 0.35 eV. P = 5 bars.

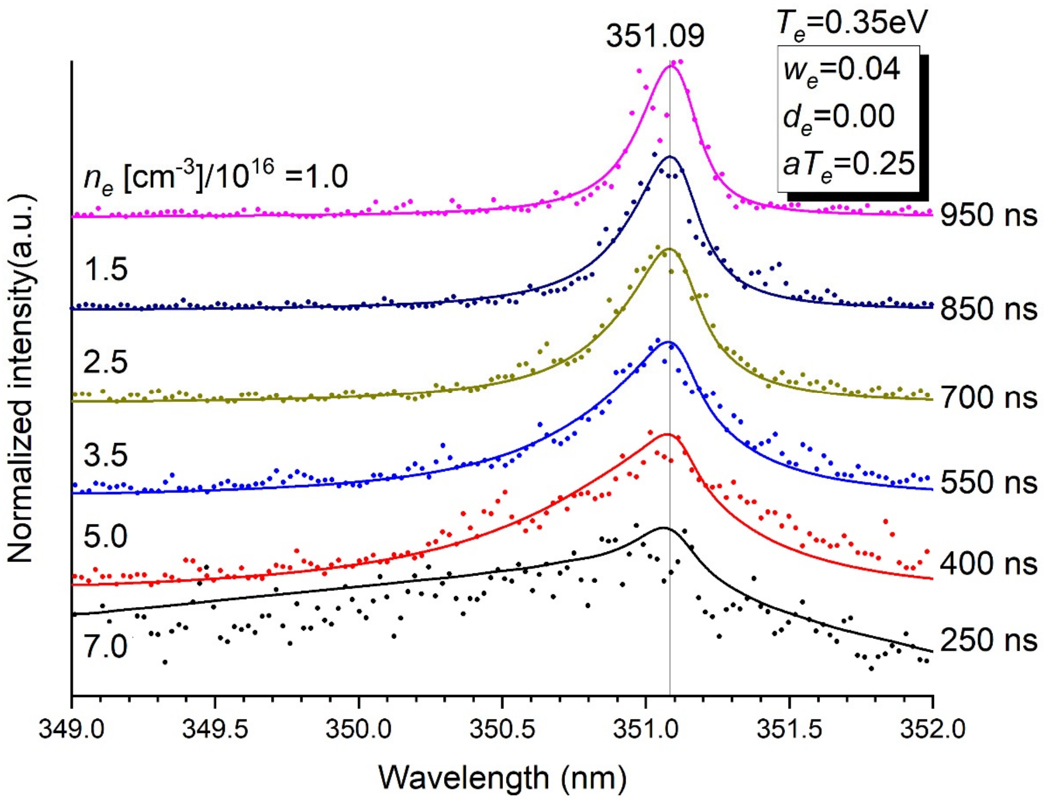

Figure S4e: Normalized intensity of the emission of the transition at 351.09 nm as a function of time. Stark parameters are given the inset. Contrary to other lines, it is the red wing of the transition that is asymmetric. The electron density n_e in [cm^{-3}]/10^{16} is determined for each time. T_e = 0.35 eV. P = 5 bars.

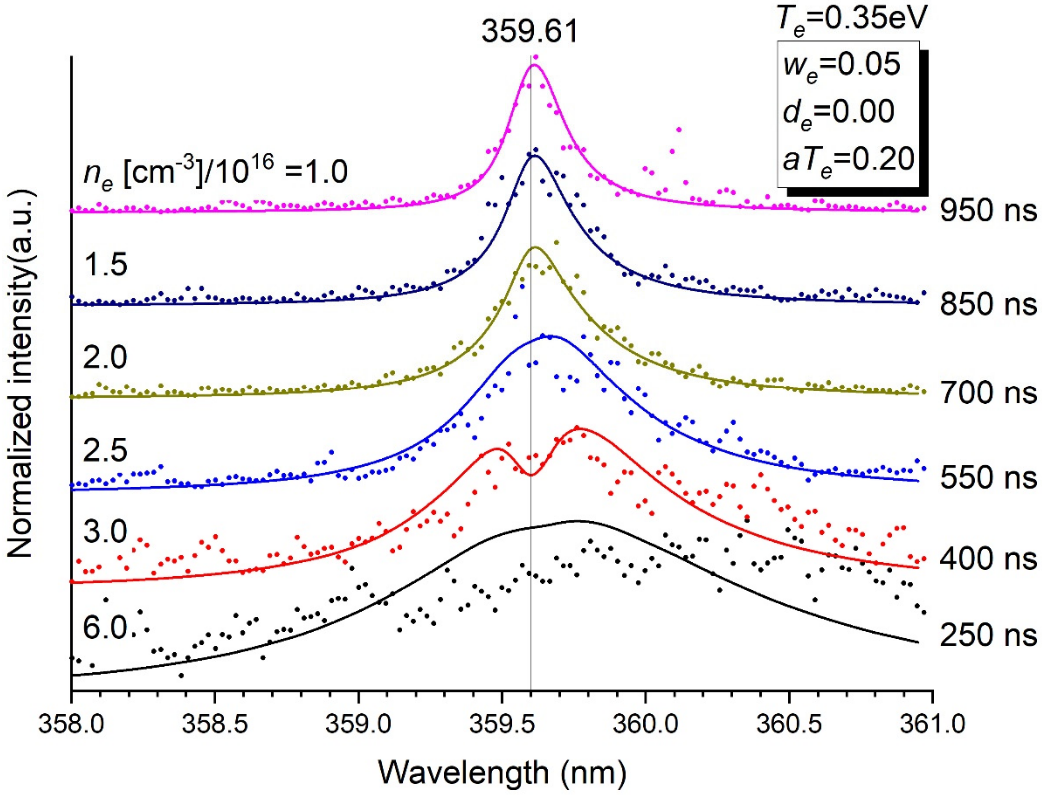

Figure S4f: Normalized intensity of the emission of the transition at 359.61 nm as a function of time. Stark parameters are given the inset. The electron density n_e in [cm^{-3}]/10^{16} is determined for each time. T_e = 0.35 eV. P = 5 bars.

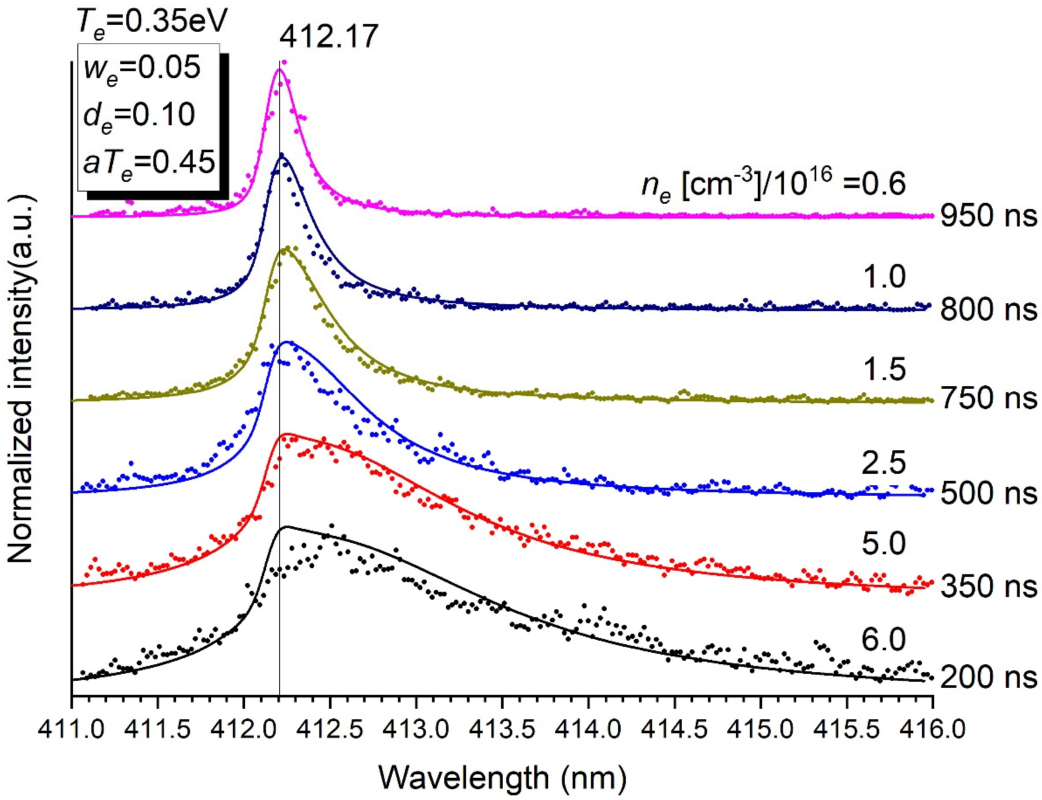

Figure S4g: Normalized intensity of the emission of the transition at 412.17 nm as a function of time. Stark parameters are given the inset. The electron density n_e in [cm^{-3}]/10^{16} is determined for each time. T_e = 0.35 eV. P = 5 bars.

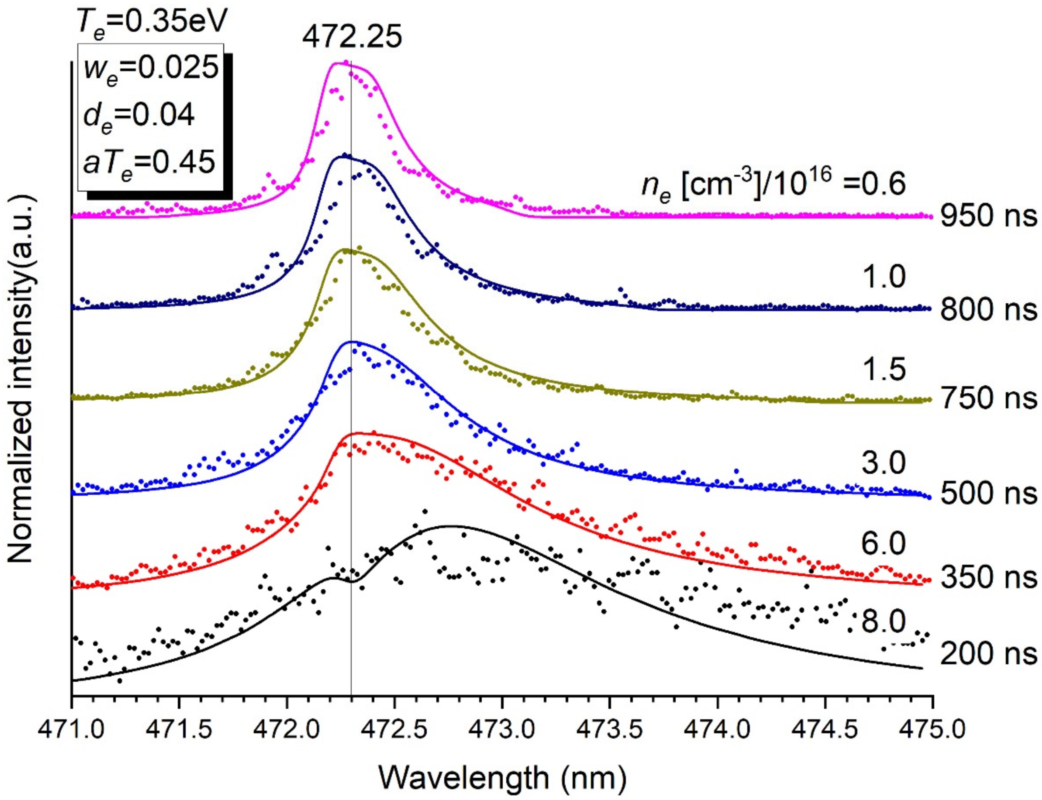

Figure S4h: Normalized intensity of the emission of the transition at 472.25 nm as a function of time. Stark parameters are given the inset. The electron density n_e in [cm^{-3}]/10^{16} is determined for each time. T_e = 0.35 eV. P = 5 bars.

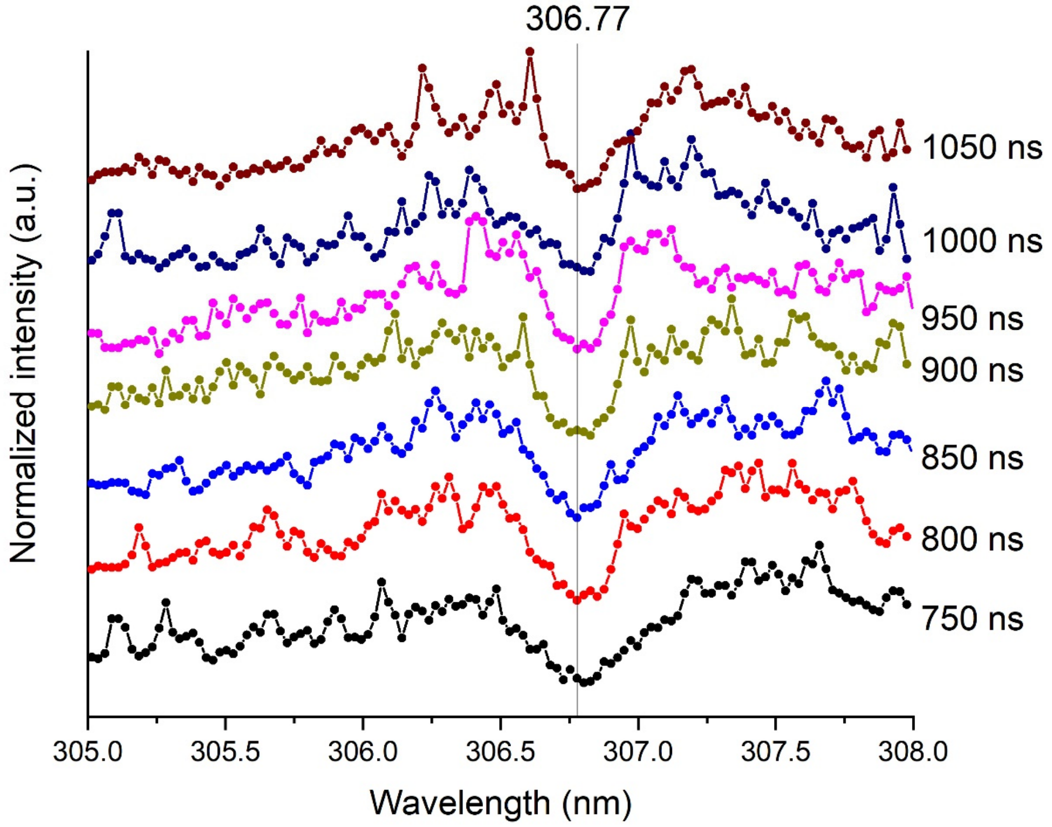

Figure S5: Detail of all experimental spectra recorded after 750 ns (where this phenomenon is the most likely–see **Figure 3** in the main text) where the possible double reversal is theoretically expected to occur (transition at 306.77 nm). The reader is invited to form his own opinion.

(A)

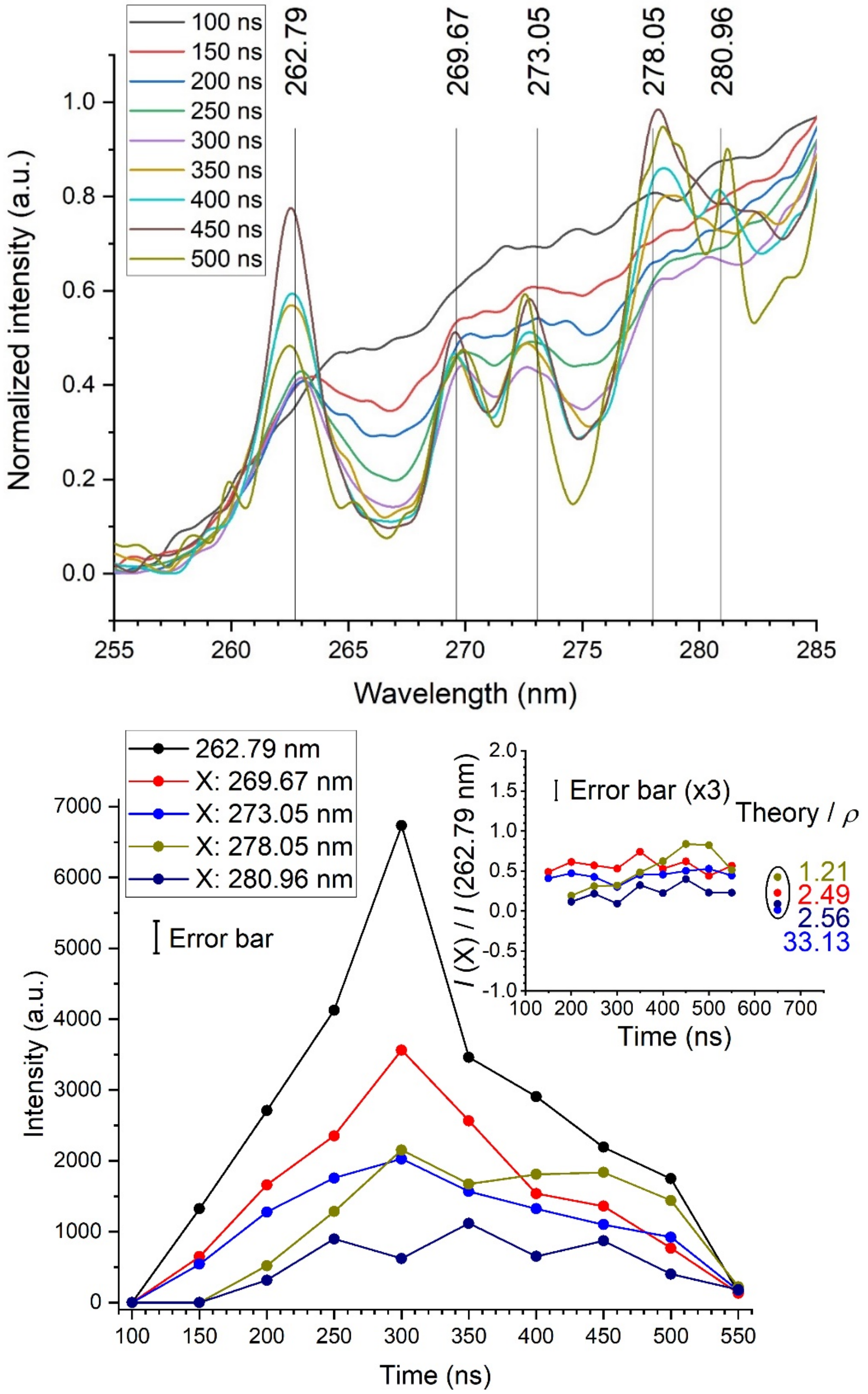

(B)

Figure S6: (**A**) Evidence of Bi I emission with normalized intensities of 1 weak (at 262.79 nm) and 4 very weak lines in the UV region. 100 gr. mm^{-1} grating. Integration time: 50 ns. These lines are too weak to be observed with the 1800 gr. mm^{-1} grating. (**B**) Time evolution of these lines (true intensities) as measured with the 100 gr. mm^{-1} grating. Insert: Time evolution of the ratio of the intensity of a given line X to the intensity of the line at 262.79 nm. “Theory” and ρ have the same meaning as in **Figure 7**.

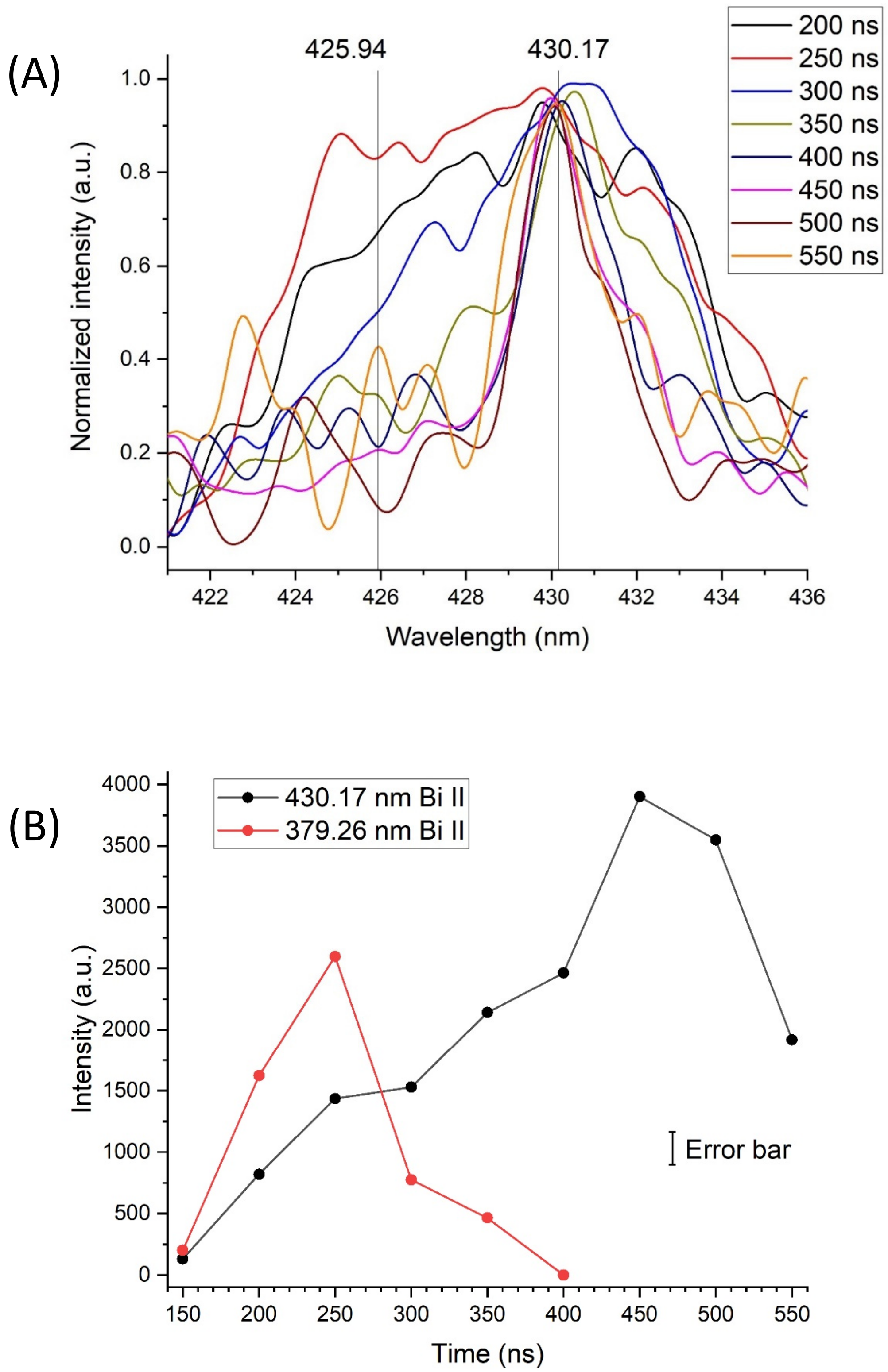

Figure S7: (**A**) Evidence of Bi II emission of 1 weak line(at 430.17 nm) in the UV region. 100 gr. mm^{-1} grating. Integration time: 50 ns. This line (and the one at 379.26 nm) are too weak to be observed with the 1800 gr. mm^{-1} grating. (**B**) Time evolution of these lines as measured with the 100 gr. mm^{-1} grating.
